# Risk factors of chronic kidney diseases in Chinese adults with type 2 diabetes

**DOI:** 10.1038/s41598-018-32983-1

**Published:** 2018-10-02

**Authors:** Lin Yang, Tsun Kit Chu, Jinxiao Lian, Cheuk Wai Lo, Pak Ki Lau, Hairong Nan, Jun Liang

**Affiliations:** 1 School of Nursing, The Hong Kong Polytechnic University, Hong Kong Special Administrative Region, China; 2 Department of Family Medicine & Primary Healthcare, New Territory West Cluster, Hospital Authority, Hong Kong Special Administrative Region, China; 3 School of Optometry, The Hong Kong Polytechnic University, Hong Kong Special Administrative Region, China; 4 Faculty of Health and Social Science, The Hong Kong Polytechnic University, Hong Kong Special Administrative Region, China

## Abstract

In this study we conducted a cross sectional study to comprehensively evaluated the risk factors of chronic kidney disease (CKD) in a large sample of Chinese adults under primary care for type 2 diabetes mellitus (T2DM). We investigated the risk factors associated with the prevalence of CKD in adults with T2DM, who were enrolled in the Risk Factor Assessment and Management Programme for Patients with Diabetes Mellitus (RAMP-DM) of Hong Kong from July 2014 to June 2017. We collected the individual data of 31,574 subjects, with mean age of 63.0 (±10.8) years and mean DM duration of 7.4 (±6.4) years. Of them 9,386 (29.7%) had CKD and 7,452 (23.6%) had micro- or macro-albuminuria. After adjustment for multiple demographic and lifestyle confounders, we identified several modifiable risk factors associated with higher rate of CKD: obesity (OR = 1.54), current smoking (OR = 1.33), higher systolic blood pressure (OR = 1.01), dyslipidemia (OR = 1.32 and 0.61 for triglycerides (TG) and high-density lipoprotein cholesterol (HDL-C)), hyperglycemia (OR = 1.11 for HbA_1c_), diabetic retinopathy (OR = 1.36 and 2.60 for non-sight and sight threatening retinopathy), and stroke (OR = 1.43). The risk factors of lower dialytic blood pressure and coronary heart disease were identified only in men, whereas peripheral arterial disease only in women. In conclusion, several modifiable and gender specific risk factors were significantly associated with higher prevalence of CKD in Chinese adults with T2DM. The high-risk populations identified in this study shall receive regular screening for renal functions to achieve better patient management in primary care settings.

## Introduction

Diabetes Mellitus (DM) is one of the most common chronic diseases worldwide. Globally, in 2014 there were 8.5% of people living with DM which nearly doubled the prevalence rate of 4.7% in 1980^1^. A clear rising trend was observed in Hong Kong in the past decade, and type 2 (T2DM) accounts for over 90% of all the persons with diabetes^2^. The International Diabetes Federation estimated that in 2011 the prevalence of diabetes was 9.4% in adults aged 20–79 years of Hong Kong, and this rate could reach 11.9% by 2030^2^.

Chronic kidney diseases (CKD) is one of common complications associated with both type 1 and type 2 DM. In the US, 40% of patients with CKD also had DM^3^. The mortality in DM complicated with CKD was estimated as high as 48 deaths per 1,000 patient-years at risk, and this rate tripled if patients also had cardiovascular conditions^3^. Previous studies have identified several risk factors for CKD, such as male, older age, obesity, hypercholesterolemia, hypertension and hyperglycemia^4–7^. However, most studies used glomerular filtration rate to identify CKD (GFR < 60 ml/min per 1.73 m^2^) or end-stage kidney disease (ESKD, GFR < 15 ml/min per 1.73 m^2^)^8^. Few have combined GFR and albuminuria categories, both of which were required for the diagnosis of CKD^9,10^. Furthermore, data are relatively limited in Chinese population under primary care.

In Hong Kong, the Risk Factor Assessment and Management Programme for Patients with Diabetes Mellitus (RAMP-DM) was implemented in the General Outpatients Clinics (GOPC) managed by the Hospital Authority (HA) since 2009, to conduct a comprehensive risk assessment and screening for diabetic complications in the primary care setting. Another objective of RAMP-DM was to educate DM patients by involving multi-disciplinary healthcare professionals including doctors, nurses and optometrists. Previous studies in Hong Kong have extensively evaluated the risks of cardiovascular diseases and diabetic retinopathy in patients of RAMP-DM^11–13^. Two recent publications have assessed the risk of developing end-stage renal disease (ESRD) among RAMP-DM participants^14,15^. Here we conducted a cross-sectional study using the RAMP-DM data, with the aim to explore the risk factors associated with CKD in Chinese adults. The literature on the risk factors of CKD is less for T2DM than for type 1, and 99% of patients registered to RAMP-DM were diagnosed with T2DM; hence, type 1 and gestational DM were excluded in this study.

## Results

### Subject characteristics

There were 40,781 people with DM enrolled into RAMP-DM of NTWC from 2 July 2014 to 16 June 2017, and 39,652 diagnosed with T2DM were eligible in this study. Of them, 8,078 (20%) subjects were further excluded from subsequent analyses because of missing data in estimated glomerular filtration rate (eGFR) and urine albumin-to-creatinine ratio (UACR). The missing data of other variables ranged 0–1.4%, with the only exception of diabetic retinopathy (DR) status, which had 4.4% and 3.3% missing in the subjects with or without CKD. The demographic characteristics of these subjects with missing data were similar to those included in the analysis (data not shown). A final sample size of 31,574 adults with T2DM were eligible for analysis. Table 1 summarizes the demographic and clinical characteristics of patients. The mean age of the study cohort is 63.0 ± 10.8 years with mean DM duration 7.4 ± 6.4 years. The number of female and male patients was nearly equal. The prevalence of CKD was 29.7% (9386/31574) at their first RAMP-DM assessment (Supplementary Table S1). 3,650 (11.6%) patients with T2DM were classified as the eGFR stages of G3a through G5, 7,452 (23.6%) as the UACR stages of A2 through A3 (micro- and macroalbuminuria), and three (<0.01%) already reached ESKD that required dialysis.

**Table 1:** Descriptive statistics of demographic and clinical characteristics of all the people with type 2 diabetes, those with and without chronic kidney disease (CKD).

Variables	Overall n = 31574	With CKD n = 9386	Without CKD n = 22188	P-value^a^
**Socio-demographic (n**, **%)**				
Sex (n, %)				<0.001
Female	15649 (49.6%)	4882 (52.0%)	10767 (48.5%)	
Male	15925 (50.4%)	4504 (48.0%)	11421 (51.5%)	
Age (Years, mean ± SD)	63.0 ± 10.8	67.0 ± 11.7	61.3 ± 9.9	<0.001
Education (n, %)				<0.001
No formal education	3993 (12.6%)	1802 (19.2%)	2191 (9.9%)	
Primary	11754 (37.2%)	3687 (39.3%)	8067 (36.4%)	
Secondary	14016 (44.4%)	3447 (36.7%)	10569 (47.6%)	
Tertiary	1682 (5.3%)	414 (4.4%)	1268 (5.7%)	
Receiving CSSA (n, %)	3018 (9.6%)	1232 (13.1%)	1786 (8.0%)	<0.001
BMI category (n, %)				<0.001
Underweight	376 (1.2%)	113 (1.2%)	263 (1.2%)	
Normal	6357 (20.1%)	1659 (17.7%)	4698 (21.2%)	
Overweight	6723 (21.3%)	1867 (19.9%)	4856 (21.9%)	
Obese	18073 (57.2%)	5718 (60.9%)	12355 (55.7%)	
Smoking (n, %)				0.010
Non-smoker	21572 (68.3%)	6319 (67.3%)	15253 (68.7%)	
Current Smoker	4402 (13.9%)	1299 (13.8%)	3103 (14.0%)	
Ex-smoker	5574 (17.7%)	1761 (18.8%)	3813 (17.2%)	
Alcohol (n, %)				<0.001
Non-drinker	23251 (73.6%)	7093 (75.6%)	16158 (72.8%)	
Current Drinker	1465 (4.6%)	399 (4.3%)	1066 (4.8%)	
Ex-drinker	1751 (5.5%)	594 (6.3%)	1157 (5.2%)	
Social drinker	4950 (15.7%)	1243 (13.2%)	3707 (16.7%)	
**Clinical characteristics**				
DM Duration (Years, mean ± SD)	7.4 ± 6.4	8.9 ± 7.1	6.7 ± 5.9	<0.001
CHD (n, %)	764 (2.4%)	315 (3.4%)	449 (2.0%)	<0.001
Stroke (n, %)	1302 (4.1%)	608 (6.5%)	694 (3.1%)	<0.001
PAD (n, %)			<0.001	
Yes	299 (0.9%)	143 (1.5%)	156 (0.7%)	
Suspected	139 (0.4%)	48 (0.5%)	91 (0.4%)	
DR Status (n, %)				<0.001
No DR	20276 (64.2%)	5041 (53.7%)	15235 (68.7%)	
Non-sight threatening	6333 (20.1%)	1990 (21.2%)	4343 (19.6%)	
Sight threatening	3131 (9.9%)	1575 (16.8%)	1556 (7.0%)	
Ungradable	90 (0.3%)	31 (0.3%)	59 (0.3%)	
**Biomedical measurements (mean** ± **SD)**				
SBP (mmHg), n = 31559	131.7 ± 16.2	134.4 ± 17.2	130.5 ± 15.5	<0.001
DBP (mmHg), n = 31559	74.8 ± 10.2	74.0 ± 10.9	75.2 ± 9.9	<0.001
UACR (mg/mmol), n = 31574	6.4 ± 26.5	18.8 ± 46.3	1.2 ± 0.6	<0.001
eGFR (ml/min/1.73 m^2^), n = 31574	84.1 ± 19.7	72.3 ± 23.1	89.0 ± 15.6	<0.001
HbA_1c_ (mmol/mol), n = 31574	54 ± 11	55 ± 13	53 ± 10	<0.001
(%)	7.1 ± 1.2	7.2 ± 1.4	7.0 ± 1.1	
TG (mmol/L), n = 31574	1.5 ± 1.0	1.6 ± 1.1	1.4 ± 0.9	<0.001
LDL-C (mmol/L), n = 31162	2.3 ± 0.7	2.3 ± 0.7	2.3 ± 0.7	0.003
HDL-C (mmol/L), n = 31572	1.3 ± 0.3	1.2 ± 0.3	1.3 ± 0.3	<0.001

^a^P-Value of Chi-square test for categorical data and of Mann-Whitney test for continuous data.

**Figure 1:**
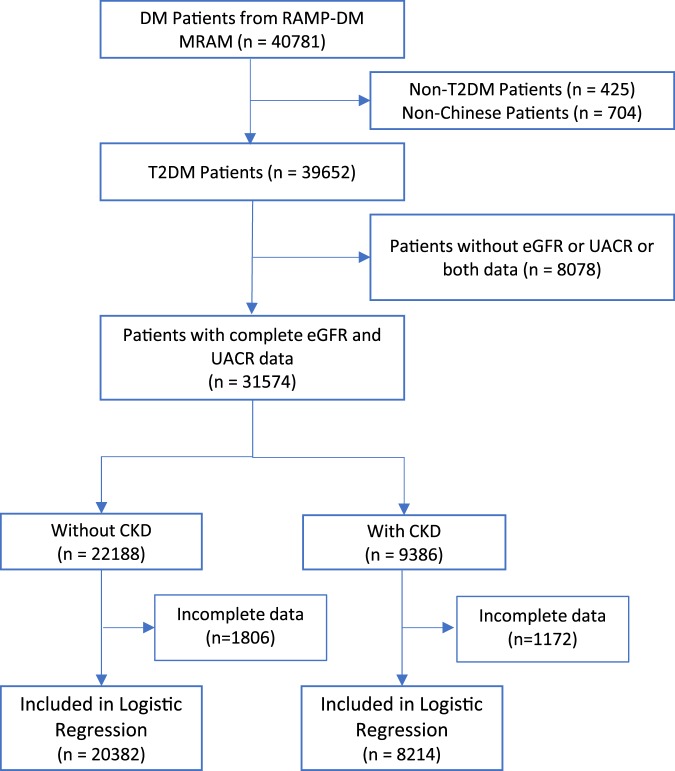
Flow chart of data extraction and analysis. Incomplete data refer to missing in at least one of the following variables: sex, age, DM duration, education, smoking, alcohol drinking, SBP, DBP, hypertension, BMI categories, CHD, stroke, PAD, DR status, HbA1c, HDL-C, LDL-C, TG, receiving CSSA.

Due to the missing data of some covariates, there were 28,596 participants included in regression analysis (Fig. 1). Women, those without former education, receiving comprehensive social security assistance (CSSA), ex-smokers, ex-/nondrinkers and obese people had a higher prevalence of CKD than the other people did. Compared to those without CKD, patients with CKD were older, had longer duration of diabetes, worse glycemic control and lipid profile (higher HbA_1c_, TG, and lower HDL-C), and higher likelihood of other diabetic complications (Table 1). Subjects with CKD were more likely to have stage 2 hypertension, with higher systolic blood pressure (SBP) but slightly lower diastolic blood pressure (DBP). The probability of receiving insulin treatment and other medications was also significantly higher in those with CKD than those without (data not shown).

### Risk factors of CKD

The crude ORs of CKD associated with different factors were estimated from univariate logistic regressions (Table 2). All the estimates were significant except for underweight (vs normal), current smokers (vs non-smokers), and suspected peripheral arterial disease (PAD) (vs no PAD). All the variables that were significant in univariate model remained significant after adjustment, but generally had smaller magnitude than the crude estimates, except for body mass index (BMI) categories, smoking, TG, and HDL-C. After adjustment, low-density lipoprotein cholesterol (LDL-C) became positively associated with CKD, but no longer significant. The association of coronary heart disease (CHD) and CKD became marginally significant with a smaller effect estimate. The adjusted OR of SBP and DBP were similar to the crude OR, with a significant inverse association found between DBP and CKD.

**Table 2:** Crude and adjusted odds ratio (OR) of chronic kidney disease (CKD) associated with different factors.

Variable	Crude OR (95% CI)^a^	*p*-value^b^	Adjusted OR (95% CI)^c^	*p*-value^b^
**Sex**: Male	0.87 (0.83, 0.91)	<0.001	0.82 (0.76, 0.88)	<0.001
**Age (Years)**	1.05 (1.05, 1.06)	<0.001	1.05 (1.04, 1.05)	<0.001
**DM Duration (Years)**	1.05 (1.05, 1.06)	<0.001	1.02 (1.02, 1.03)	<0.001
**Education**				
Primary	0.56 (0.52, 0.60)	<0.001	0.86 (0.79, 0.94)	0.001
Secondary	0.40 (0.37, 0.43)	<0.001	0.83 (0.76, 0.92)	<0.001
Tertiary	0.40 (0.35, 0.45)	<0.001	0.81 (0.69, 0.94)	0.006
**BMI category**				
Underweight	1.22 (0.97, 1.52)	0.091	1.28 (0.97, 1.68)	0.071
Overweight	1.09 (1.01, 1.18)	0.031	1.15 (1.05, 1.25)	0.003
Obesity	1.31 (1.23, 1.40)	<0.001	1.54 (1.42, 1.66)	<0.001
**Smoking**				
Current smoker	1.01 (0.94, 1.08)	0.773	1.33 (1.22, 1.46)	<0.001
Ex-smoker	1.11 (1.05, 1.19)	<0.001	1.10 (1.01, 1.20)	0.023
**Alcohol drinking**				
Current drinker	0.85 (0.76, 0.96)	0.008	/	
Social drinker	0.76 (0.71, 0.82)	<0.001	/	
Ex-drinker	1.17 (1.05, 1.30)	0.003	/	
**Receiving CSSA**	1.73 (1.60, 1.86)	<0.001	1.23 (1.12, 1.35)	<0.001
**CHD**	1.68 (1.45, 1.95)	<0.001	1.17 (0.99, 1.39)	0.068
**Stroke**	2.15 (1.92, 2.40)	<0.001	1.43 (1.26, 1.63)	<0.001
**PAD**				
Suspected	1.26 (0.88, 1.78)	0.198	1.12 (0.75, 1.65)	0.572
Yes	2.19 (1.74, 2.75)	<0.001	1.66 (1.28, 2.16)	<0.001
**DR Status**				
Non-sight threatening	1.38 (1.30, 1.47)	<0.001	1.36 (1.27, 1.46)	<0.001
Sight threatening	3.06 (2.83, 3.30)	<0.001	2.60 (2.38, 2.84)	<0.001
Ungradable	1.59 (1.02, 2.44)	0.038	1.08 (0.67, 1.72)	0.740
**SBP**	1.01 (1.01, 1.02)	<0.001	1.01 (1.01, 1.01)	<0.001
**DBP**	0.99 (0.99, 0.99)	<0.001	0.99 (0.99, 0.99)	<0.001
**Hypertension**				
Elevated	1.24 (1.15, 1.34)	<0.001	/	
Stage 1	1.20 (1.12, 1.29)	<0.001	/	
Stage 2	1.65 (1.54, 1.77)	<0.001	/	
**HbA**_**1c**_	1.17 (1.14, 1.19)	<0.001	1.11 (1.09, 1.14)	<0.001
**TG**	1.21 (1.18, 1.24)	<0.001	1.32 (1.27, 1.38)	<0.001
**LDL-C**	0.96 (0.92, 0.99)	0.016	1.03 (0.99, 1.07)	0.152
**HDL-C**	0.51 (0.47, 0.55)	<0.001	0.61 (0.55, 0.68)	<0.001

Reference level for sex = female, education = no formal education, BMI category = normal, smoking = non-smoker, alcohol drinking = non-drinker, receiving CSSA = no, hypertension = normal, CHD = no, stroke = no, PAD = no, DR status = no.

^a^Crude OR estimated from the univariate logistic regression models.

^b^p-value of Wald test for individual factors.

^c^Adjusted OR estimated from the stepwise multivariate logistic regression model with all the above variables added except hypertension stages. The variable of alcohol drinking was eliminated from the final model during stepwise selection.

### Sensitivity analysis

We did a sensitivity analysis by replacing the continuous variables SBP and DBP with hypertension stages, which gave similar effect estimates to the main analysis for all the covariates (Supplementary Table S2). The adjusted OR showed an increasing trend over hypertension stages, but no significant difference was found between elevated and normal stages. We also filled in the missing values of LDL-C by multiple imputation and the estimates were similar to those from main analysis (Supplementary Table S2).

### Stratified analysis by gender

We compared the characteristics of subjects with or without CKD for men and women, respectively (Supplementary Table S3). Among the subjects with CKD, compared to men, women were older, living longer with DM, less likely obese or having other diabetic complications, with lower levels of UACR and eGFR. The gender difference in HbA_1c_, TG, LDL-C and HDL-C was small, although all reached statistical significance. Similar patterns were found in male and female subjects with CKD.

The interaction terms of gender with BMI categories, DR status and HbA_1c_ were significant (data not shown); we therefore did a stratified analysis of female and male subjects. The effect estimates after adjustment were similar between female and male, except education, DBP, CHD and BMI categories (Supplementary Table S4). An inverse association between DBP and CKD, as well as a positive association between CHD and CKD, were found significant in men but not in women. PAD was associated with a higher rate of CKD, but only significant in women.

## Discussion

We conducted a large-scale cross-sectional study in 31,574 participants with individual medical records in primary care setting, to identify the risk factors of CKD and potential gender differences. We found that the prevalence of micro- and macro-albuminuria in Chinese adults with T2DM was 23.6%, which is within the range of prevalence rates reported in a global cross sectional study (9.8–38.8%)^16^, and also comparable to the rates of 28.9% and 25.2% that were reported by two cross-sectional studies in mainland China^6,7^. The prevalence of CKD defined by both eGFR and albuminuria was 29.7% in our subjects, which is also close to the prevalence rate of 27.1% in another study of Chinese population^7^ but slightly lower than the rates (34.5–36.4%) in the U.S.^17^. It has been under debate that eGFR may not accurately reflect the real renal function of T2DM adults^18^. Our study found that the prevalence of CKD dramatically decreased if only eGFR or albuminuria criteria were applied (29.7% vs 11.6% and 23.6%). This highlights the need of incorporating routine urine albumin tests into the management program of persons with diabetes for early detection of CKD.

It is surprising that in our study men had a lower rate of CKD than women, which is contrary to the previous findings^4^. This could be explained by the fact that female subjects in our study were older and had a longer duration of DM than males. The stratified analysis identified some common risk factors of CKD shared by both female and male, and gender-specific factors such as DBP and CHD that were only significant in men, and PAD only in women. Gender difference has been well documented in the previous studies on ESRD and mortality in populations with diabetes^19,20^. Our findings further support that different prediction models shall be separately established for women and men.

The association of CHD and CKD has been widely demonstrated^21^. This association is particularly strong in persons with diabetes, as both are caused by accumulating damages to vascular systems during the disease progress^22^. We found that the CKD subjects were more likely to have CHD as compared to those without CKD. Similarly, several cross-sectional studies in Chinese adults with T2DM also demonstrated significant association of CHD with CKD^23,24^. However, we could not establish the temporal sequence of these adverse events due to the limitation of cross-sectional study design. In a cohort study of T2DM subjects in Hong Kong, CHD was associated with a higher risk of CKD^25^. It will be of great interest to conduct prospective cohort studies with the aim to explore the possibility of using CHD as an early predictor for CKD events or vice versa.

The results from the multivariate model showed that SBP was significantly associated with CKD, whereas an inverse association was found between DBP and CKD. This finding echoes a study that were conducted in mainland China with smaller sample size, which also found that SBP, retinopathy, TC, TG and anemia were risk factors of CKD^7^. In sensitivity analysis, we adopted the new AHA classification of hypertension and found that compared to subjects with normal BP, those at hypertension stage 1 and 2 had a significantly higher prevalence of CKD, but no significant difference was found between normal and elevated groups. This finding is consistent with one previous study in mainland China that found hypertension could significantly increase the risk of CKD^5^.

There are several limitations in this study. First, we used a cross-sectional study design, therefore could not establish the causal relationship between risk factors and CKD. Nevertheless, a large sample size still allowed us to have statistical power large enough to identify the significant risk factors for CKD. The findings of this study could also shed light on future cohort studies when more follow-up data become available. Second, it was uncertain that CKD in our subjects was diabetic nephropathy, since they might also have abnormal kidney structures or other diseases that deposited them to a higher risk of CKD. Third, in this study we used creatinine-based UACR estimates which have been criticized for lack of accuracy especially within the normal or high range of GFR^10^. Nevertheless, creatinine-based UACR is still widely adopted in current clinical practice. Fourth, this study population were the Chinese adults enrolled in NTWC; hence, the findings might not be applicable to all the Chinese populations. Last but not least, although we have adjusted for multiple variates in regression models, there were potential confounding factors that were not routinely collected in clinical practice still remained unadjusted.

In conclusion, we found a high prevalence rate of CKD in Chinese adults with T2DM in this large-scale cross-sectional study. We also identified several modifiable risk factors associated with CKD, including obesity, current smoking, higher SBP, lower DBP (only in men), dyslipidemia, hyperglycemia, the presence of diabetic retinopathy, stroke, PAD (only in women) and CHD (only in men). Given the increasing trend of T2DM prevalence and ageing population, the high-risk populations identified in this study shall receive regular screening for renal functions to achieve better patient management in primary care settings. Future studies on risk or beneficial factors of CKD incidence and disease progress could be conducted in this large patient cohort when more follow-up data become available, in order to achieve better prognosis for Chinese adults with diabetes.

## Methods

### Data sources

Individual data of people who were enrolled in the RAMP-DM from 2 July 2014 to 16 June 2017 were obtained from the New Territories West Cluster (NTWC) of the Hospital Authority (HA). The study period was chosen because of the good data completeness. This cluster serves the population of 1.09 million living in the northwest region of Hong Kong Special Administrative Region. Participation into RAMP-DM is voluntary. After 2014, over 90% of people with diabetes have been enrolled to RAMP-DM (Jun Liang, data not published). The details of RAMP-DM have been described elsewhere^11,26^. Briefly, individual data of demographics, education, lifestyle (smoking, alcohol and physical activity), family history, blood pressure, body mass index (BMI) and waist-hip ratio (WHR) were collected during their first visits to clinics. The participants were assessed for diabetic neurological, macrovascular and microvascular complications, coronary heart disease, stroke, malignancy and diabetic retinopathy. Fasting blood samples were taken during each visit to test for HbA_1c_, fasting glucose, total cholesterol, LDL-C, HDL-C, triglyceride (TG) and serum creatinine. Urine samples were also taken to test for albumin to calculate urine albumin to creatinine ratio (UACR), urine protein to creatinine ratio (PCR). The estimated glomerular filtration rate (eGFR) was calculated using the following formula^27^:

$${\rm{eGFR}}=175\ast {(\frac{\mathrm{serum}\mathrm{creatinine}}{88.4})}^{-1.154}\ast ag{e}^{-0.203}\ast (0.742\,if\,female)$$

Individual data collected at their first RAMP-DM assessment were analyzed in this study. Additional laboratory data of eGFR, UACR and HbA_1c_ during hospitalized episodes and outpatient visits were retrieved from the Clinical Data Analysis and Reporting System (CDARS) for the period of 180 days before to 180 days after the first RAMP-DM assessment, by matching the unique patient reference numbers. The laboratory data on the dates closest to the first RAMP-DM assessment were selected for subsequent analyses.

### Chronic kidney diseases

The CKD diagnosis followed the 2012 Kidney Disease Improving Global Outcomes (KDIGO) guideline^28^, which combines eGFR and UACR to define CKD. The eGFR stages include G1 normal and high (≥90 ml/min/1.73 m^2^), G2 mild reduction (60–89), G3a mild-moderate reduction (45–59), G3b moderate-severe reduction (30–44), G4 severe reduction (15–29) and G5 kidney failure (<15). The UACR stages are A1 normal to miLDL-Cy increased (<3 mg/mmol), A2 moderately increased (microalbuminuria) (3–30) and A3 severely increased (macroalbuminuria) (>30). CKD is defined as the eGFR stages of G3a through G5 and/or the UACR stages of A2 through A3.

### Risk factors and covariates

Individual demographic data, such as age, gender, duration of T2DM, occupation, education and whether receiving Comprehensive Social Security Assistance (CSSA), were included in the model as potential risk factors. Smoking status was classified into current, ex- and non-smokers. Alcohol drinking was grouped into current, social, ex- and non-drinkers. Duration of DM was the number of years between the first RAMP-DM assessment date and the first year of DM diagnosis. Obesity status was categorized according to the Asian criteria: underweight as BMI < 18.5, normal as 18.5 ≤ BMI < 23, overweight as 23 ≤ BMI < 25, and obese as BMI ≥ 25. Individual data of blood pressure will be included in the model as continuously variables or alternatively as categorical variable indicating the level of hypertension status. Hypertension status of each subject was classified by systolic blood pressure (SBP) and diastolic blood pressure (DBP) that were measured at their first assessment in RAMP-DM, according to the 2017 Guidelines for High Blood Pressure in Adults from the American College of Cardiology/American Heart Association^29^. Patients are classified into four categories: normal (SBP < 120 mmHg and DBP < 80 mmHg), elevated (120 mmHg ≤ SBP < 130 mmHg and DBP < 80 mmHg), stage 1 (130 mmHg ≤ SBP < 140 mmHg or 80 mmHg ≤ DBP < 90 mmHg), and stage 2 (SBP ≥ 140 mmHg or DBP ≥ 90 mmHg). The diagnosed diabetic complications of coronary heart disease (CHD), stroke, peripheral arterial disease (PAD) were also retrieved for the first RAMP-DM assessment. Diabetic retinopathy was classified into four categories: (1) sight threatening retinopathy were those who had laser, vitrectomy or anti-vascular endothelia growth factor (anti-VEGF) therapy, or were classified as severe non-proliferative DR, proliferative DR or maculopathy); (2) non sight threatening retinopathy were those who are classified as mild/moderate non-proliferative DR, and no maculopathy and no laser for both eyes; (3) No DR are those who were classified as no-DR without any maculopathy, laser, vitrectomy or anti-VEGF therapy; (4) Ungradable were those with at least one eye classified as “Not known” grade of DR, maculopathy and laser, and not classified as severe pre-proliferative DR, proliferative DR, and also without vitrectomy nor anti-VEGF therapy. We also retrieved lipid profiles of TG, HDL-C and LDL-C cholesterol measured at the first RAMP-DM assessment for all the subjects. Individual records of HbA_1c_, eGFR and UACR were retrieved from the electronic health records of CDARS by matching the unique patient reference numbers.

### Statistical analysis

The participants with and without CKD were compared for their demographic and clinical characteristics using Chi-square test for categorical variables and t-test for continuous variables (normally distributed) or Mann-Whitney (not normally distributed). Each variable was assessed for proportion of missing data, and the subjects with complete and incomplete data were compared for other variables, in order to determine whether data imputation was necessary. Univariate logistic regressions were performed to identify the potential covariates and multivariate logistic regression to estimate the effects after adjustment for covariates. We fit a stepwise multivariate logistic regression model, which adjusted for sex, age, DM duration, education, BMI, smoking, alcohol drinking, receiving CSSA, CHD, stroke, PAD, DR status, HbA_1c_, TG, LDL-C, HDL-C, and both SBP and DBP as continuous variables. Only alcohol drinking was eliminated from the final multivariate model. Adjusted odd ratios (OR) and the corresponding 95% confidence interval (CI) were calculated for the variables in the regression model. The product variables of gender with other significant covariates were respectively added into interaction models to assess the gender difference. If any significance was found in these interaction terms, stratified analysis by gender was also conducted to estimate the age-specific effect estimates. Statistical analysis was conducted by using R version 3.4.2 (The R Foundation for Statistical Computing). A *P*-value less than 0.05 was considered as statistically significant.

### Sensitivity analysis

We replaced the linear covariates of SBP and DBP by hypertension categories defined according to the new AHA classification of hypertension^29^. We did another sensitivity analysis by filling the missing values of LDL-C with multiple imputation on age, sex, DM duration, BMI, HDL-C, TG and comorbidity, and the above multivariate regression was repeated using the new dataset.

### Ethical approval and informed consent

The ethical approval has been obtained from the New Territory West Cluster Clinical and Research Ethics Committee. All research was performed in accordance with relevant guidelines. Consent forms were exempted because all the data were extracted from the computerized data system of Hospital Authority, and no personal data were collected for this study.

## Electronic supplementary material

Supplementary file

## Data Availability

The data that support the findings of this study are available from the Hospital Authority of Hong Kong Special Administrative Region, but restrictions apply to the availability of these data, which were used under license for the current study, and so are not publicly available. Data are however available from the authors upon reasonable request and with permission of the Hospital Authority of Hong Kong Special Administrative Region.
