# Supplementary material for: Risk factors of chronic kidney diseases in Chinese adults with type 2 diabetes
Source: Sci Rep. 2018 Oct 2;8:14686. doi: 10.1038/s41598-018-32983-1 (PMC6168551; doi:10.1038/s41598-018-32983-1)
Supplement: Supplementary file 1 — Supplementary file [file 41598_2018_32983_MOESM1_ESM.docx]

**Risk factors of chronic kidney diseases in Chinese adults with type 2 diabetes**

**Lin YANG^1^, Tsun Kit CHU^2^, Jinxiao LIAN^3^, CW LO^2^, Pak Ki LAU^1^, Hairong NAN^4^, Jun LIANG^2*^**

^1^ School of Nursing, The Hong Kong Polytechnic University, Hong Kong Special Administrative Region

^2^ Department of Family Medicine & Primary Healthcare, New Territory West Cluster, Hospital Authority, Hong Kong Special Administrative Region

^3^ School of Optometry, The Hong Kong Polytechnic University, Hong Kong Special Administrative Region

^4^ Faculty of Health and Social Science, The Hong Kong Polytechnic University, Hong Kong Special Administrative Region

*Corresponding author: Dr Jun Liang, Department of Family Medicine & Primary Healthcare, NTW Cluster, Hospital Authority, Hong Kong Special Administrative Region. Tel: +852 2468 6090 Fax: +852 2468 6651 Email: [liangj@ha.org.hk](mailto:liangj@ha.org.hk)

Supplementary Table S1. Distribution of the estimated glomerular filtration rate (eGFR) and urine albumin-to-creatinine ratio (UACR) categories in subjects.

|  |  | UACR Categories | | |  |
| --- | --- | --- | --- | --- | --- |
|  |  | A1 | A2 | A3 | Subtotal |
| eGFR Categories | G1 | 9988 | 2072 | 197 | 12257 |
|  | G2 | 12200 | 2947 | 520 | 15667 |
|  | G3a | 1574 | 834 | 273 | 2681 |
|  | G3b | 334 | 328 | 170 | 832 |
|  | G4 | 26 | 50 | 58 | 134 |
|  | G5 |  | 1 | 2 | 3 |
|  | Subtotal | 24122 | 6232 | 1220 | 31574 |

Supplementary Table S2. Sensitivity analysis: crude and adjusted odds ratio (OR) of chronic kidney disease (CKD) associated with different factors.

| **Variable** | **Sensitivity analysis 1^a^** | | **Sensitivity analysis 2^b^** | |
| --- | --- | --- | --- | --- |
|  | **Adjusted OR (95% CI)** | ***p*-value^c^** | **Adjusted OR**  **(95% CI)** | ***p*-value^c^** |
| **Sex**: Male | 0.79  (0.74, 0.85) | <0.001 | 0.81  (0.75, 0.86) | <0.001 |
| **Age (Years)** | 1.05  (1.05, 1.05) | <0.001 | 1.05  (1.04, 1.05) | <0.001 |
| **DM Duration (Years)** | 1.03  (1.02, 1.03) | <0.001 | 1.02  (1.02, 1.03) | <0.001 |
| **Education** |  |  |  |  |
| Primary | 0.86  (0.79, 0.94) | 0.001 | 0.86  (0.79, 0.94) | <0.001 |
| Secondary | 0.83  (0.75, 0.91) | <0.001 | 0.83  (0.75, 0.91) | <0.001 |
| Tertiary | 0.80  (0.69, 0.94) | 0.005 | 0.80  (0.69, 0.94) | 0.005 |
| **BMI category** |  |  |  |  |
| Underweight | 1.28  (0.98, 1.67) | 0.074 | 1.29  (0.99, 1.69) | 0.063 |
| Overweight | 1.16  (1.06, 1.27) | 0.001 | 1.16  (1.06, 1.27) | 0.001 |
| Obesity | 1.57  (1.45, 1.69) | <0.001 | 1.57  (1.45, 1.69) | <0.001 |
| **Smoking** |  |  |  |  |
| Current smoker | 1.34  (1.23, 1.47) | <0.001 | 1.34  (1.22, 1.47) | <0.001 |
| Ex-smoker | 1.09  (1.01, 1.19) | 0.036 | 1.09  (1.01, 1.19) | 0.032 |
| **Receiving CSSA** | 1.22  (1.12, 1.34) | <0.001 | 1.22  (1.12, 1.34) | <0.001 |
| **CHD** | 1.18  (1.00, 1.40) | 0.056 | 1.17  (0.99, 1.39) | 0.064 |
| **Stroke** | 1.42  (1.25, 1.62) | <0.001 | 1.43  (1.25, 1.62) | <0.001 |
| **PAD** |  |  |  |  |
| Suspected | 1.08 (0.73, 1.60) | 0.705 | 1.11  (0.75, 1.64) | 0.618 |
| Yes | 1.68  (1.30, 2.18) | <0.001 | 1.68  (1.29, 2.18) | <0.001 |
| **DR Status** |  |  |  |  |
| Non-sight threatening | 1.37  (1.28, 1.47) | <0.001 | 1.36  (1.27, 1.45) | <0.001 |
| Sight threatening | 2.64  (2.42, 2.88) | <0.001 | 2.58  (2.36, 2.81) | <0.001 |
| Ungradable | 1.04  (0.65, 1.66) | 0.870 | 1.06  (0.66, 1.69) | 0.806 |
| **SBP** | / |  | 1.01  (1.01, 1.01) | <0.001 |
| **DBP** | / |  | 0.99  (0.99, 1.00) | <0.001 |
| **Hypertension** |  |  |  |  |
| Elevated | 1.08  (0.99, 1.18) | 0.093 | / |  |
| Stage 1 | 1.09  (1.01, 1.18) | 0.037 | / |  |
| Stage 2 | 1.30  (1.20, 1.41) | <0.001 | / |  |
| **HbA_1c_** | 1.12  (1.09, 1.15) | <0.001 | 1.12  (1.10, 1.15) | <0.001 |
| **TG** | 1.21  (1.18, 1.25) | <0.001 | 1.21  (1.18, 1.25) | <0.001 |
| **LDL-C** | 1.04  (1.00, 1.09) | 0.0403 | 1.04  (1.00, 1.08) | 0.0718 |
| **HDL-C** | 0.58  (0.53, 0.65) | <0.001 | 0.59  (0.53, 0.65) | <0.001 |

Reference level for sex=female, education=no formal education, BMI category=normal, smoking=non-smoker, alcohol drinking=non-drinker, receiving CSSA=no, hypertension=normal, CHD=no, stroke=no, PAD=no, DR status=no.

^a^ Sensitivity analysis 1: Adjusted ORs were estimated from the stepwise multivariate logistic regression model with all the above variables and hypertension stages.

^b^ Sensitivity analysis 2: The stepwise multivariate logistic regression model that contains all the above variables and SBP/DBP was fitted to the dataset with missing data of LDL-C filled in by multiple imputation.

^c^ P-value of Wald test for individual factors.

Supplementary Table S3. Comparison of characteristics between male and female subjects.

| **Variables** | **With CKD** | |  | **Without CKD** | |  |  |
| --- | --- | --- | --- | --- | --- | --- | --- |
|  | **Male**  **(n=4504)** | **Female**  **(n=4882)** | **P-value^a^** | **Male**  **(n=11421)** | **Female**  **(n=10767)** | **P-value^a^** |  |
| **Socio-demographic (n, %)** | | | |  |  |  |  |
| Age (Years, mean ± SD) | 65.7 ± 11.3 | 68.3 ± 11.8 | <0.001 | 61.0 ± 9.7 | 61.6 ± 10.1 | 0.002 |  |
| Education (n, %) |  |  | <0.001 |  |  | <0.001 |  |
| No formal education | 358 (7.9%) | 1444 (29.6%) |  | 489 (4.3%) | 1702 (15.8%) |  |  |
| Primary | 1724 (38.3%) | 1963 (40.2%) |  | 3609 (31.6%) | 4458 (41.4%) |  |  |
| Secondary | 2090 (46.4%) | 1357 (27.8%) |  | 6327 (55.4%) | 4242 (39.4%) |  |  |
| Tertiary | 311 (6.9%) | 103 (2.1%) |  | 945 (8.3%) | 323 (3.0%) |  |  |
| Receiving CSSA (n, %) | 533 (11.8%) | 699 (14.3%) | <0.001 | 840 (7.4%) | 946 (8.8%) | <0.001 |  |
| BMI category (n, %) |  |  | 0.001 |  |  | <0.001 |  |
| Underweight | 35 (0.8%) | 78 (1.6%) |  | 109 (1.0%) | 154 (1.4%) |  |  |
| Normal | 767 (17.0%) | 892 (18.3%) |  | 2241 (19.6%) | 2457 (22.8%) |  |  |
| Overweight | 903 (20.0%) | 964 (19.7%) |  | 2644 (23.2%) | 2212 (20.5%) |  |  |
| Obese | 2788 (61.9%) | 2930 (60.0%) |  | 6419 (56.2%) | 5936 (55.1%) |  |  |
| Smoking (n, %) |  |  | <0.001 |  |  | <0.001 |  |
| Non-smoker | 1799 (39.9%) | 4520 (92.6%) |  | 5157 (45.2%) | 10096 (93.8%) |  |  |
| Current Smoker | 1144 (25.4%) | 155 (3.2%) |  | 2766 (24.2%) | 337 (3.1%) |  |  |
| Ex-smoker | 1560 (34.6%) | 201 (4.1%) |  | 3492 (30.6%) | 321 (3.0%) |  |  |
| Alcohol (n, %) |  |  | <0.001 |  |  | <0.001 |  |
| Non-drinker | 2581 (57.3%) | 4512 (92.4%) |  | 6366 (55.7%) | 9792 (90.9%) |  |  |
| Current Drinker | 366 (8.1%) | 33 (0.7%) |  | 993 (8.7%) | 73 (0.7%) |  |  |
| Ex-drinker | 514 (11.4%) | 80 (1.6%) |  | 990 (8.7%) | 167 (1.6%) |  |  |
| Social drinker | 1019 (22.6%) | 224 (4.6%) |  | 3034 (26.6%) | 673 (6.3%) |  |  |
|  |  |  |  |  |  |  |  |
| **Clinical characteristics** |  |  |  |  |  |  |  |
| DM Duration (Years, mean ± SD) | 8.6 ± 6.9 | 9.3 ± 7.4 | <0.001 | 6.5 ± 5.7 | 6.9 ± 6.0 | <0.001 |  |
| CHD (n, %) | 170 (3.8%) | 145 (3.0%) | 0.093 | 279 (2.4%) | 170 (1.6%) | <0.001 |  |
| Stroke (n, %) | 299 (6.6%) | 309 (6.3%) | 0.764 | 415 (3.6%) | 279 (2.6%) | <0.001 |  |
| PAD (n, %) |  |  | 0.022 |  |  | 0.001 |  |
| Yes | 78 (1.7%) | 65 (1.3%) |  | 105 (0.9%) | 51 (0.5%) |  |  |
| Suspected | 19 (0.4%) | 29 (0.6%) |  | 44 (0.4%) | 47 (0.4%) |  |  |
| DR Status (n, %) |  |  | <0.001 |  |  | <0.001 |  |
| No DR | 2309 (51.3%) | 2732 (56.0%) |  | 7611 (66.6%) | 7624 (70.8%) |  |  |
| Non-sight threatening | 1023 (22.7%) | 967 (19.8%) |  | 2463 (21.6%) | 1880 (17.5%) |  |  |
| Sight threatening | 825 (18.3%) | 750 (15.4%) |  | 830 (7.3%) | 726 (6.7%) |  |  |
| Ungradable | 16 (0.4%) | 15 (0.3%) |  | 33 (0.3%) | 26 (0.2%) |  |  |
|  |  |  |  |  |  |  |  |
| **Biomedical measurements (mean ± SD)** | | | |  |  |  |  |
| SBP (mmHg) | 133.8 ± 16.9 | 135.0 ± 17.5 | 0.006 | 130.5 ± 15.0 | 130.5 ± 16.1 | 0.261 |  |
| DBP (mmHg) | 75.0 ± 11.0 | 73.1 ± 10.7 | <0.001 | 76.3 ± 9.9 | 73.9 ± 9.8 | <0.001 |  |
| UACR (mg/mmol) | 21.2 ± 50.9 | 16.7 ± 41.6 | <0.001 | 1.1 ± 0.6 | 1.3 ± 0.6 | <0.001 |  |
| eGFR (ml/min/1.73m^2^) | 72.9 ± 50.9 | 71.7 ± 22.9 | 0.075 | 89.0 ± 15.9 | 89.0 ± 15.3 | 0.644 |  |
| HbA_1c_ (mmol/mol) | 56 ± 13 | 55 ± 12 | <0.001 | 53 ± 11 | 53 ± 10 | 0.183 |  |
| (%) | 7.3 ± 1.4 | 7.2 ± 1.3 |  | 7.0 ± 1.2 | 7.0 ± 1.1 |  |  |
| TG (mmol/L) | 1.6 ± 1.2 | 1.6 ± 1.1 | <0.001 | 1.4 ± 1.0 | 1.4 ± 0.9 | <0.001 |  |
| LDL-C (mmol/L) | 2.3 ± 0.7 | 2.3 ± 0.7 | <0.001 | 2.2 ± 0.6 | 2.3 ± 0.7 | <0.001 |  |
| HDL-C (mmol/L) | 1.1 ± 0.3 | 1.3 ± 0.3 | <0.001 | 1.2 ± 0.3 | 1.4 ± 0.3 | <0.001 |  |

^a^ P-Value of Chi-square test for categorical data and of Mann-Whitney test for continuous data.

Supplementary Table S4. Stratified analysis by gender.

| **Variable** | **Male** | | **Female** | |
| --- | --- | --- | --- | --- |
|  | **Adjusted OR (95% CI)** | **p-value^a^** | **Adjusted OR (95% CI)** | **p-value^a^** |
| Age | 1.04 (1.04, 1.05) | <0.001 | 1.05 (1.05, 1.06) | <0.001 |
| Education |  |  |  |  |
| Primary | 0.82 (0.69, 0.97) | 0.023 | 0.87 (0.78, 0.97) | 0.010 |
| Secondary | 0.74 (0.62, 0.88) | <0.001 | 0.89 (0.79, 1.00) | 0.060 |
| Tertiary | 0.73 (0.58, 0.91) | 0.006 | 0.86 (0.66, 1.11) | 0.259 |
| Receiving CSSA | 1.18 (1.03, 1.35) | 0.019 | 1.27 (1.12, 1.43) | <0.001 |
| BMI cateogory |  |  |  |  |
| Underweight | 1.15 (0.72, 1.77) | 0.551 | 1.37 (0.96, 1.92) | 0.075 |
| Overweight | 1.04 (0.92, 1.19) | 0.520 | 1.25 (1.10, 1.41) | <0.001 |
| Obese | 1.46 (1.30, 1.64) | <0.001 | 1.59 (1.44, 1.77) | <0.001 |
| Smoking |  |  |  |  |
| Current smoker | 1.30 (1.17, 1.44) | <0.001 | 1.47 (1.16, 1.84) | 0.001 |
| Ex-smoker | 1.08 (0.98, 1.18) | 0.130 | 1.27 (1.02, 1.57) | 0.033 |
| Alcohol |  |  |  |  |
| Current drinker | 0.93 (0.80, 1.08) | 0.353 | 0.93 (0.57, 1.49) | 0.784 |
| Ex-drinker | 1.04 (0.90, 1.18) | 0.610 | 0.89 (0.64, 1.22) | 0.475 |
| Social drinker | 0.91 (0.83, 1.00) | 0.059 | 0.92 (0.77, 1.09) | 0.326 |
| DM Duration | 1.03 (1.02, 1.03) | <0.001 | 1.02 (1.02, 1.03) | <0.001 |
| CHD | 1.28 (1.02, 1.60) | 0.028 | 1.04 (0.79, 1.35) | 0.786 |
| Stroke | 1.30 (1.09, 1.55) | 0.004 | 1.61 (1.33, 1.96) | <0.001 |
| PAD |  |  |  |  |
| Yes | 1.37 (0.97, 1.91) | 0.071 | 2.20 (1.44, 3.38) | <0.001 |
| Suspected | 1.08 (0.58, 1.91) | 0.808 | 1.15 (0.67, 1.94) | 0.607 |
| DR Status |  |  |  |  |
| Non-sight threatening | 1.41 (1.29, 1.56) | <0.001 | 1.32 (1.20, 1.46) | <0.001 |
| Sight threatening | 2.91 (2.58, 3.29) | <0.001 | 2.30 (2.02, 2.61) | <0.001 |
| Ungradable | 1.26 (0.64, 2.39) | 0.481 | 0.94 (0.46, 1.83) | 0.850 |
| SBP | 1.01 (1.01, 1.02) | <0.001 | 1.01 (1.01, 1.01) | <0.001 |
| DBP | 0.99 (0.98, 0.99) | <0.001 | 1.00 (0.99, 1.00) | 0.156 |
| HbA_1c_ | 1.14 (1.10, 1.18) | <0.001 | 1.08 (1.05, 1.12) | <0.001 |
| TG | 1.32 (1.25, 1.40) | <0.001 | 1.32 (1.25, 1.41) | <0.001 |
| LDL-C | 1.02 (0.96, 1.09) | 0.485 | 1.04 (0.98, 1.10) | 0.209 |
| HDL-C | 0.56 (0.48, 0.66) | <0.001 | 0.65 (0.57, 0.75) | <0.001 |

Reference level for education=no formal education, BMI category=normal, smoking=non-smoker, alcohol drinking=non-drinker, receiving CSSA=no, hypertension=normal, CHD=no, stroke=no, PAD=no, DR status=no.

^a^ P-value of Wald test for individual factors
